# Study of the characteristics and properties of the SiO_2_/TiO_2_/Nb_2_O_5_ material obtained by the sol–gel process

**DOI:** 10.1038/s41598-020-80310-4

**Published:** 2021-01-13

**Authors:** Bruna Teixeira da Fonseca, Eliane D’Elia, José Márcio Siqueira Júnior, Sanair Massafra de Oliveira, Kelly Leite dos Santos Castro, Emerson Schwingel Ribeiro

**Affiliations:** 1 Instituto de Química, Universidade Federal Do Rio de Janeiro - UFRJ, CT, Bloco A, Cidade Universitária – Ilha do Fundão, Rio de Janeiro, RJ CEP 21941-909 Brazil; 2 Instituto de Química, Universidade Federal Fluminense – UFF, Valonguinho, Niterói, RJ CEP 24020-150 Brazil

**Keywords:** Materials chemistry, Chemical synthesis

## Abstract

The SiO_2_/TiO_2_/Nb_2_O_5_ material was set by the sol–gel method and was characterized by several techniques through thermogravimetric, spectroscopic, and textural analyzes. For the two synthesized materials, the specific surface area was 350.0 and 494.0 m^2^ g^−1^ (SiTiNb-A and SiTiNb-B, respectively). An enhance of the crystalline order with the temperature increase of the thermal treatment was observed. Through X-ray Photoelectron Spectroscopy analysis, the binding energy values for the Ti 2p and Nb 3d levels showed the insertion of Ti and Nb atoms in the silica matrix. The Electron Dispersive Spectroscopy analyses also confirmed the high dispersion of the metals presented on the materials surface. The Thermogravimetric Analysis showed weight loss for the of 37.6% (SiTiNb-A) and 29.7% (SiTiNb-B). The presence of the crystalline phases TiO_2_-anatase and monoclinic-Nb_2_O_5_ in the materials was confirmed through the data obtained by association of powder X-ray Diffraction and FT-Raman. Values obtained from optical band-gap aimed the dependence of the oxides concentration and the calcination temperature. Finally, the pyridine adsorption studies have indicated the presence of Lewis and Brønsted acid sites.

## Introduction

The sol–gel process is a synthesis methodology widely used to obtain inorganic materials, as it is based on the growth of a three-dimensional polymeric silica network, which may or may not be modified by the insertion of other metals in the network, adding new characteristics and properties and allowing applications of these materials in various areas. The sol–gel process has been widely applied in the synthesis of mixed oxides for being a simple and reproducible methodology. Thus, several research groups employ the sol–gel process to obtain oxide mixtures of the type SiO_2_/M_x_O_y_ or SiO_2_/M_x_O_y_/N_x_O_y_, where M_x_O_y_ or N_x_O_y_ are metallic oxides dispersed in the silica matrix such as: Al_2_O_3,_ TiO_2_, WO_3_, Nb_2_O_5_, MoO_3_, CeO_2_, CaO, Sb_2_O_5_ and others^1–10^.

The great importance of the sol–gel process is because of the different characteristics and properties obtained by the synthesis of mixed oxides which are unique. The characteristics of the sol–gel process are well known: the reactions operate at low temperatures obtaining materials with high thermal stability, high purity, controlled porosity and high uniformity for the distribution of the metal oxides in the silica matrix (M_x_O_y_ or N_x_O_y_) and increasing the number of active sites on the surface. Besides this, these materials combine the mechanical properties of the silica matrix with the chemical properties of the metal oxides, exhibiting different chemical and physical properties from those observed in the oxide alone, especially because the presence of Brønsted and Lewis acid sites on the oxide mixture^3,4,11–14^. Hence, the oxides mixture are interesting materials for various applications^3,4^: as heterogeneous catalysis^2,9,15,16^, photoanodes for solar cells^6^, photocatalytic activity^7^, optical properties^8^, bioactive glass^10^, preconcentration systems of metallic ions^11,17–19^, dye remediation or adsorbent in aqueous solution^5,20^, adsorption of interest molecules^21^, development of new chemically modified electrodes^22,23^, among others.

Recently, Xu et al.^24^ exhibited the assembly of SiO_2_/Nb_2_O_5_/TiO_2_–SiO_2_ broadband antireflective coating by the sol–gel process. Firstly, there was the preparation of the Nb_2_O_5_ composite sol, and apart, the preparation of TiO_2_–SiO_2_ composite sol to get finally triple-layer broadband antireflective coatings. The methodology by Xu et al. is used to obtain mixed oxides in the shape of films; otherwise from obtaining mixed oxides in the form of micrometric materials with large surface area and porosity, as previously presented.

Differently from Xu et al., the article researching group has contributed to the development and applications of new micrometric ternary oxides obtained by sol–gel process. Among the applications, it can be highlighted that the development of new chemically modified electrodes^22,23,25,26^ and the preconcentration of metallic ions systems^11,17,27–29^. Based on that, the main purpose of this work is to obtain and characterize SiO_2_/TiO_2_/Nb_2_O_5_ ternary oxides obtained by the sol–gel process as well to study its properties, for further applications.

## Materials and methods

### Reagents and solutions

The tetraethylorthosilicate (TEOS, 98%), titanium (IV) butoxide (97%) and niobium (V) pentachloride (99%) were purchased from Sigma-Aldrich (Saint Louis, MO, USA). Ethanol (99.8%), HCl (37% v/v) and HNO_3_ (95% v/v) were purchased from Vetec (Duque de Caxias, RJ, Brazil). Ultra-pure Milli-Q water was used (resistivity > 18.2 MΩ cm^−1^, 25 °C, Millipore Milli-Q purification system, Billerica, MA, USA).

### Synthesis of the SiO_2_/TiO_2_/Nb_2_O_5_ material

The SiO_2_/TiO_2_/Nb_2_O_5_ material (designed by SiTiNb) was prepared in two mass proportions, and the samples obtained were SiTiNb-A and SiTiNb-B. The choice theoretical mass percent of TiO_2_ and Nb_2_O_5_ on the materials was based on other studies of the group, to verify this dependence in the properties, such as acidic properties, specific surface area, optical properties, among others. The synthesis procedure for SiTiNb-A material was: 230.0 mL of a 50% (v/v) ethanol/TEOS solution and 7.0 mL of a 6.0 mol L^−1^ HCl solution were added to a 500.0 mL reactor. The mixture was stirred for 3 h at 353 K to initiate the pre-hydrolysis of the TEOS. After that, 22.0 mL of titanium (IV) butoxide dissolved in 50.0 mL of ethanol were added drop by drop to the mixture, then more 7.0 mL of a 6.0 mol L^−1^ HCl solution. The mixture was stirred for 1 h at 353 K. In a third stage, 35.0 g of niobium (V) pentachloride dissolved in 50.0 mL of ethanol were added drop by drop and the mixture was stirred for 30 min at 353 K. After, 15.0 mL of a 6.0 mol L^−1^ HCl solution were added, and the mixture was stirred at 353 K until the formation of the gel. The resulting mixture was transferred to a beaker glass and heated at 363 K until complete evaporation of the solvent and then heated for 4 h in an oven at 363 K to form the xerogel. The obtained product (xerogel) was precipitated and then dried under vacuum (10^–5^ Pa) at 363 K for 6 h. The resulting particles were washed with ethanol in a Soxhlet extractor for 24 h to remove any metallic oxide that was not incorporated into the silica matrix, precursors residues and possible soluble species. Then, SiTiNb-A material was washed with 100.0 mL of a 0.1 mol L^−1^ HNO_3_ solution, followed by some ethanol, ultra-pure water, and ethanol again. Finally, the ternary oxide was dried under vacuum (10^–5^ Pa) for 6 h at 363 K and stored.

The same synthesis procedure for SiTiNb-B material was adopted but using the following amounts of reagents: 230.0 mL of a 50% (v/v) ethanol/TEOS solution; 45.0 mL of titanium (IV) butoxide dissolved in 50.0 mL of ethanol and 25.0 g of niobium (V) pentachloride dissolved in 50.0 mL of ethanol.

### Instrumentation and characterization

The amounts of TiO_2_ and Nb_2_O_5_ in the silica matrix were determined by using Wavelength Dispersive X-ray Fluorescence (WDXRF) on a Bruker AXS equipment (Tokyo, Japan), model S4 Pioneer, operating with Rh tube (E Kα1 = 20.2 keV).

The crystallinity of the thermally treated SiTiNb-A and SiTiNb-B materials was analyzed by powder X-ray Diffraction (XRD). A Bruker AXS D8 Advance instrument (Cu Kα1,α2 radiation, 40.0 kV and 40.0 mA) operating in a Bragg– Brentano θ/θ configuration. The diffraction patterns were collected in a flat geometry with steps of 0.02° and accumulation time of 3 s per step. Finally, the X-ray powder diffraction data were refined following the Rietveld Method with the TOPAS Academic version 5.0 software (Copyright 1992–2012 Alan A. Coelho. Where, for the activation of the program, DLL files are released by Alan).

The analysis of the Specific Surface Area (S_BET_) of the SiTiNb-A and SiTiNb-B materials was determined by using the BET (Brauner, Emmett and Teller) multipoint method on a Quantachrome Model Nova 1200e (Boynton Beach, USA) instrument. The samples were previously activated at 393 K in vacuum for 24 h and the samples with granulometry of 150.0 μm ≤ x ≤ 300.0 μm were used. The BJH (Barrett–Joyner–Halenda) method was used to obtain the average pore size and volume.

The Thermogravimetric Analysis was performed on a Shimadzu DTG-60 (Kyoto-Japan). The analyzes, in the range of 293 to 1173 K and the temperature scan rate of 5 K min^−1^, were performed with argon flow rate of 50.0 mL min^−1^.

Scanning Electron Microscopy (SEM) images were acquired using a JEOL model JSM 6460-LV (Tokyo, Japan) or FEI model Magellan 400 HXR scanning electron microscope at an acceleration voltage of 3.0 kV, 5.0 kV or 10.0 kV according to the analyzed ternary oxide, and 500 × or 1000 × of magnification. For Electron Dispersive Spectroscopy (EDS) analyses, the EDS Noran System Six model 200 (Waltham, USA) was linked to JSM 6460-LV equipment. For the SEM and EDS analyses, the sample was dispersed over double-sided conductive tape on a copper support and coated with gold before the experiment. For this analysis, the materials with a fraction of particle size less than granulometry of 90 μm were used.

The X-ray Photoelectron Spectroscopy (XPS) analysis was performed on the Esca Plus equipment from Omicron Nanotechnology instrument (Germany), in ultra-high vacuum (pressure: 10^–9^ mbar), using an Mg X-ray source (Κα = 1253.6 eV). The adjusted emission current was of 16 mA at a voltage of 12.5 kV. Survey spectra were obtained with 50.0 eV analyzer pass energy and 0.5 eV step size. The high-resolution spectra were obtained with 40.0 eV pass energy in the analyzer and 0.08 eV steps. The binding energies were referred to the carbon 1 s of high oriented pyrolytic graphite (HOPG) level, set as 284.6 eV. The peak fitting was performed using the CasaXPS Software Version 2.3.13 (License holder: **INMETRO, 25250-020 Xerem Duque de Caxias RJ, Brazil**).

The SiTiNb-A and SiTiNb-B materials samples were analyzed by powder XRD, FT-RAMAN scattering analyzes and Diffuse Reflectance Spectroscopy (DRS) in the UV–Visible region. For the analyses, the samples were thermally treated, separately, at temperatures of 473, 673, 873, 1073 and 1273 K for 8 h.

Solid-state FT-Raman spectra of the thermally treated SiTiNb-A and SiTiNb-B materials were recorded by a Bruker MultiRam spectrometer (Tokyo, Japan) at room temperature with a germanium detector, maintained at liquid nitrogen temperature. For the measurements, 1064 nm Nd–YAG laser line was used with a resolution of 2 cm^−1^ in the region of 1200–70 cm^−1^ and 256 scans at a laser power of 100.0 mW. The samples were measured in the hemispheric bore of an aluminum sample holder.

The value determination of optical band-gap of the thermally treated samples was performed through the Kubelka–Munk function (F(R)) of data interpretation from the DRS and using the best function applicable to each curve according to the software. The measurements were performed on a Cary 5000 Varian UV–Vis–NIR Spectrophotometer, with wavelengths between 190 and 950 nm and magnesium oxide as reference.

The acidic properties of the SiTiNb-A and SiTiNb-B materials were verified using pyridine as a probe molecule to detect Lewis and Brønsted acid sites in the samples. Drops of pyridine were added in the samples and they were dried under high vacuum at different temperatures (room temperature, 373 and 423 K). After dried, the samples were analyzed by Infrared Spectroscopy, obtained with pellets containing 12% of the material and 88% of KBr.

## Results and discussions

### Structural characterization

The XRF analysis was carried to access whether the synthesis procedure was efficient to obtain the oxide mixture^4^. The theoretical mass percent of TiO_2_ and Nb_2_O_5_ in the SiTiNb-A material are 10.0 and 30.0 wt.%; and for SiTiNb-B material are 20.0 and 20.0 wt.%. The results obtained for the TiO_2_ and Nb_2_O_5_ amounts in percentage by mass (wt.%) incorporated in the silica matrix, were 4.8 wt.% of TiO_2_ and 18.4 wt.% of Nb_2_O_5_ for SiTiNb-A material and 9.3 wt.% of TiO_2_ and 11.6 wt.% of Nb_2_O_5_ for SiTiNb-B material. Regarding the synthesis, the method showed a lower percentage of titanium oxide and niobium oxide after washing with ethanol in a Soxhlet apparatus with 0.1 mol L^−1^ HNO_3_ solution. These values are lower than expected probably due to the reaction medium pH and other factors, which were not favorable to the complete hydrolysis of titanium (IV) butoxide and niobium (V) pentachloride precursors.

As previously reported by Teixeira et al., it is extremely difficult to obtain SiO_2_/M_x_O_y_/N_x_O_y_ type materials in the desired proportions considering the differences between the hydrolysis kinetics of the precursors in the same reaction media^4^. In addition to that, it can generate soluble species in the washing process, for example. However, the values obtained are satisfactory to employ the SiTiNb-A and SiTiNb-B materials, and the sol–gel process is a method extremely useful and reproductive for the SiO_2_/M_x_O_y_/N_x_O_y_ type materials synthesis^4^.

**Figure 1:**
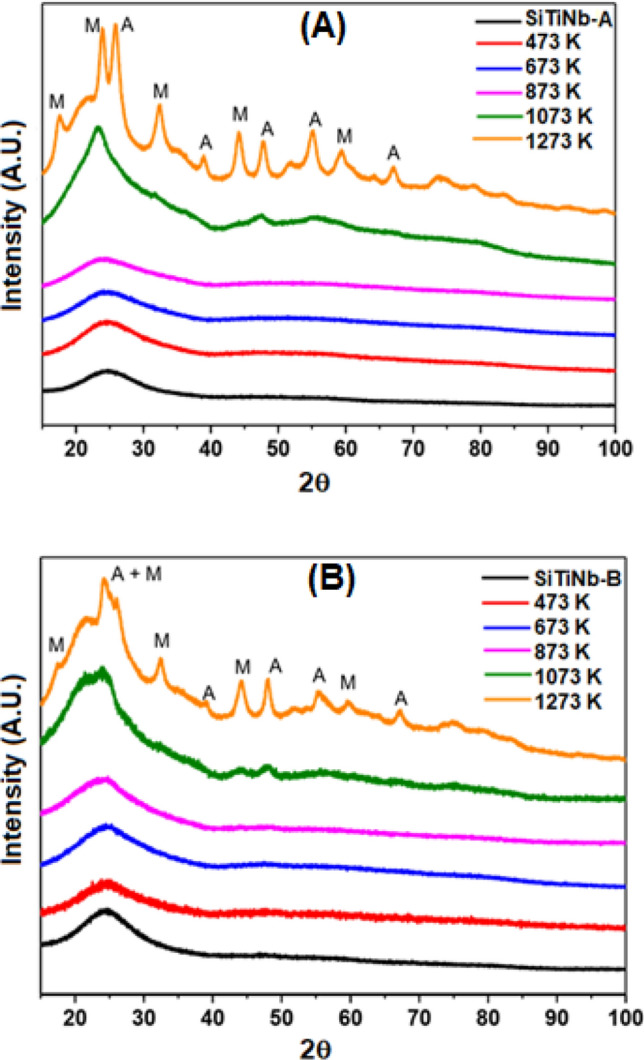
X-ray diffractograms for the SiTiNb-A **(A)** and SiTiNb-B **(B)** materials thermally treated in the range of 473–1273 K. The peaks marked with the letters A and M in the graph of samples heated to 1273 K indicate the indexed phases of TiO_2_ Anatase (A) and monoclinic Nb_2_O_5_ (M).

The Fig. 1 shows the diffractograms obtained after heat treatment at different temperatures for the SiTiNb-A (Fig. 1A) and SiTiNb-B (Fig. 1B) materials. It is observed that both materials, up to a temperature of 873 K do not present enough structural order to be observed peaks in the diffractograms and present low crystallinity, showing a peak for 2θ = 23° (around) due to the amorphous halo typical of glassy silica^30^. However, after 1073 K some peaks begin to appear without having a good definition. At a temperature of 1273 K, can be seen some peaks with greater definition, where the SiTiNb-A sample presents the highest crystallinity at this temperature. Through the values of 2θ of the peaks presented in the diffractograms, when comparing with the ICSD database, it can be identified that beyond the anatase phase of TiO_2_ ICSD#92,363 of space group I41/amd Z (141) (a = 3.7710(9) Å ; b = 3.7710(9) Å c = 9.430(2) Å; α = β = γ = 90°)^31^, there is the presence of monoclinic Nb_2_O_5_ ICSD#25,750 from space group C 12/m1 (12) (a = 28.51 Å; b = 3.83 Å; c = 17.48 Å; α = 90°; β = 120.8°; γ = 90°)^32^. A halo between 20° and 27° is also observed in 2θ indicating the presence of SiO_2_, as previously mentioned. As it will be seen under by analysis of Electron Dispersive Spectroscopy images (EDS), a high dispersion of metal oxides in the silica matrix is observed and it can be attributed to the strong interactions with siloxane groups forming strong covalent bonds. Also, there is a concentration below 10.0 wt.% of TiO_2_ in both materials. This high dispersion and low concentration of TiO_2_ in the silica matrix decreases the mobility TiO_2_ in the silica matrix, making it difficult in the formation of Anatase phase or Rutile at lower temperatures.

The Tables 1 and 2 summarize the crystallographic data obtained using the cell parameter values, crystallite size referring to TiO_2_ in the anatase phase and monoclinic-Nb_2_O_5_ phase obtained with the refinement by the Rietveld Method. In the case of SiTiNb samples, by virtue of their low crystallinity, it was only possible to perform the refinement for the diffractograms of the samples calcined from 1073 K. The Gof (Good of fitness) and Rwp (weighted profile R-factor) values were lower and better than those obtained in the refinement of samples containing niobium, showing a good approximation of the theoretical and experimental diffractograms.

**Table 1:** Crystallographic data of the SiTiNb-A samples thermally treated at 1073 and 1273 K.

Fase	Sample	a (Å)	b (Å)	c (Å)	Vol (Å^3^)	Rwp (%)	Gof	Size (nm)
TiO_2_	SiTiNb-A-1073	3.884549	3.651779	9.922338	140.753	1.16	1.06	3.34
	SiTiNb-A-1273	3.734697	3.653955	10.01743	136.702	1.63	1.55	6.80
Nb_2_O_5_	SiTiNb-A-1073	27.69484	3.832431	16.85335	1537.99	1.16	1.06	2.67
	SiTi**S**b-A-1273	27.93331	3.782507	16.72712	1515.21	1.63	1.55	7.07

**Table 2:** Crystallographic data of the SiTiNb-B samples thermally treated at 1073 and 1273 K.

Fase	Sample	a (Å)	b (Å)	c (Å)	Vol (Å^3^)	Rwp (%)	Gof	Size (nm)
TiO_2_	SiTiNb-B-1073	4.017652	3.607377	10.44006	151.310	2.64	1.03	4.62
	SiTi**S**b-B-1273	3.953890	3.551600	10.57239	148.464	1.65	1.39	9.91
Nb_2_O_5_	SiTi**N**b-B-1073	28.17731	3.726620	16.58537	1491.48	2.64	1.03	3.17
	SiTiNb-B-1273	28.21406	3.727744	16.56584	1491.83	1.65	1.39	6.51

It can be observed that, with the increase in the calcination temperature, there is an increment in the size of the crystallite, while the volume of the unit cell undergoes a small reduction, which is characteristic of the grow in the structural ordering of the sample. However, in the SiTiNb-B sample, there is a slight gain in the volume of the unit cell of the sample calcined at 1073 K in relation to that calcined at 1273 K, indicating a higher amount of anatase sites in relation to monoclinic niobium oxide may be the cause of this reduction (Tables 1, 2, 3).

**Table 3:** Percentage between the crystalline phases in the calcined SiTiNb samples.

Sample	% TiO_2_ anatase	% Nb_2_O_5_ monoclinic
SiTiNb-A-1073	16,1	83,9
SiTiNb-A-1273	32,3	67,7
SiTiNb-B-1073	7,9	92,1
SiTiNb-B-1273	27,9	72,1

According to refinement, the relative percentage between the anatase and monoclinic phases varies as specified by Table 3. It is possible to observe that the calcination temperature increase favors the anatase-TiO_2_ phase and decreases the percentage of the monoclinic-Nb_2_O_5_ phase. This behavior occurs in the same proportions in the two samples, demonstrating that it is only an effect of temperature and it is not related to the amount of metal oxide in the matrix of each material.

The BET analyses showed that the specific surface area was 494.0 m^2^ g^−1^ for SiTiNb-A and 350.0 m^2^ g^−1^ for SiTiNb-B. The BJH method revealed an average pore volume of 0.272 cm^−3^ g^−1^ for SiTiNb-A and 0.194 cm^−3^ g^−1^ for SiTiNb-B; and the average pore size of 11.0 Å for SiTiNb-A and 11.1 Å for SiTiNb-B, which indicates that these ternary oxides are microporous. These values are important, as it shows that the active sites are accessible in the SiTiNb-A and SiTiNb-B materials.

**Figure 2:**
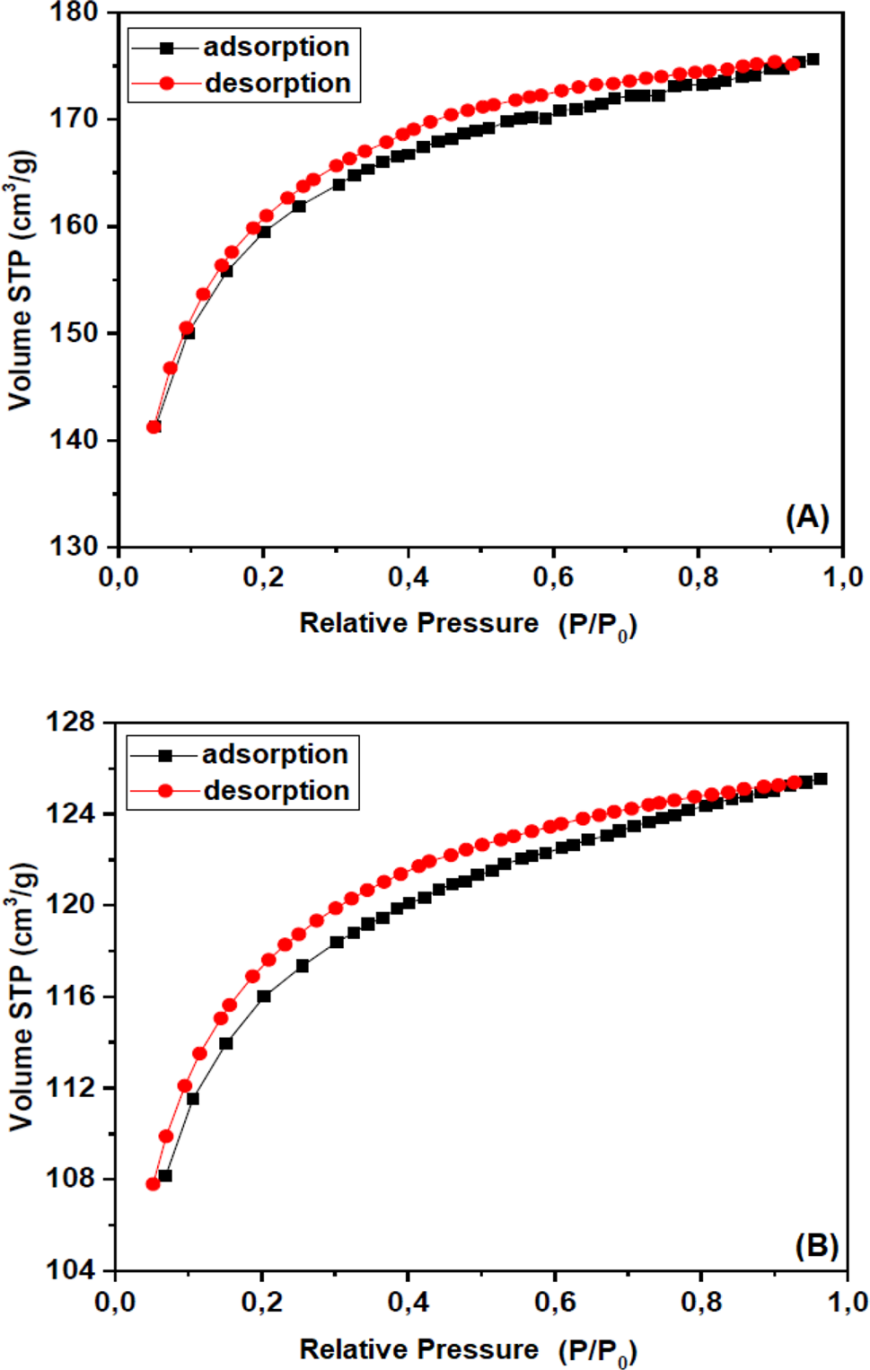
Nitrogen adsorption–desorption isotherm of SiTiNb-A **(A)** and SiTiNb-B **(B)** ternary oxides.

It can be seen by the adsorption and desorption curves (Fig. 2) for both materials, that the isotherms are type I(b)^33^, presenting a characteristic curve of microporous materials with a small external surface area^34,35^. In such cases, it is common for adsorption to occur on the accessible pore volume and not on the internal surface area. There is a small separation between the adsorption and desorption curves, pointing out a possible H4-type hysteresis^33^, which is normally associated with adsorption in micropores and characteristic of non-linear slits^36^. However, the BET analyses demonstrate a lower surface area and pore volume for SiTiNb-B, showing that the concentration of TiO_2_ and Nb_2_O_5_ oxides in the silica matrix had a greater influence on the parameters (surface area and pore volume) and probably hardens the structure due to the formation of a dense layer and/or the coalescence of pores^7,8^.

**Figure 3:**
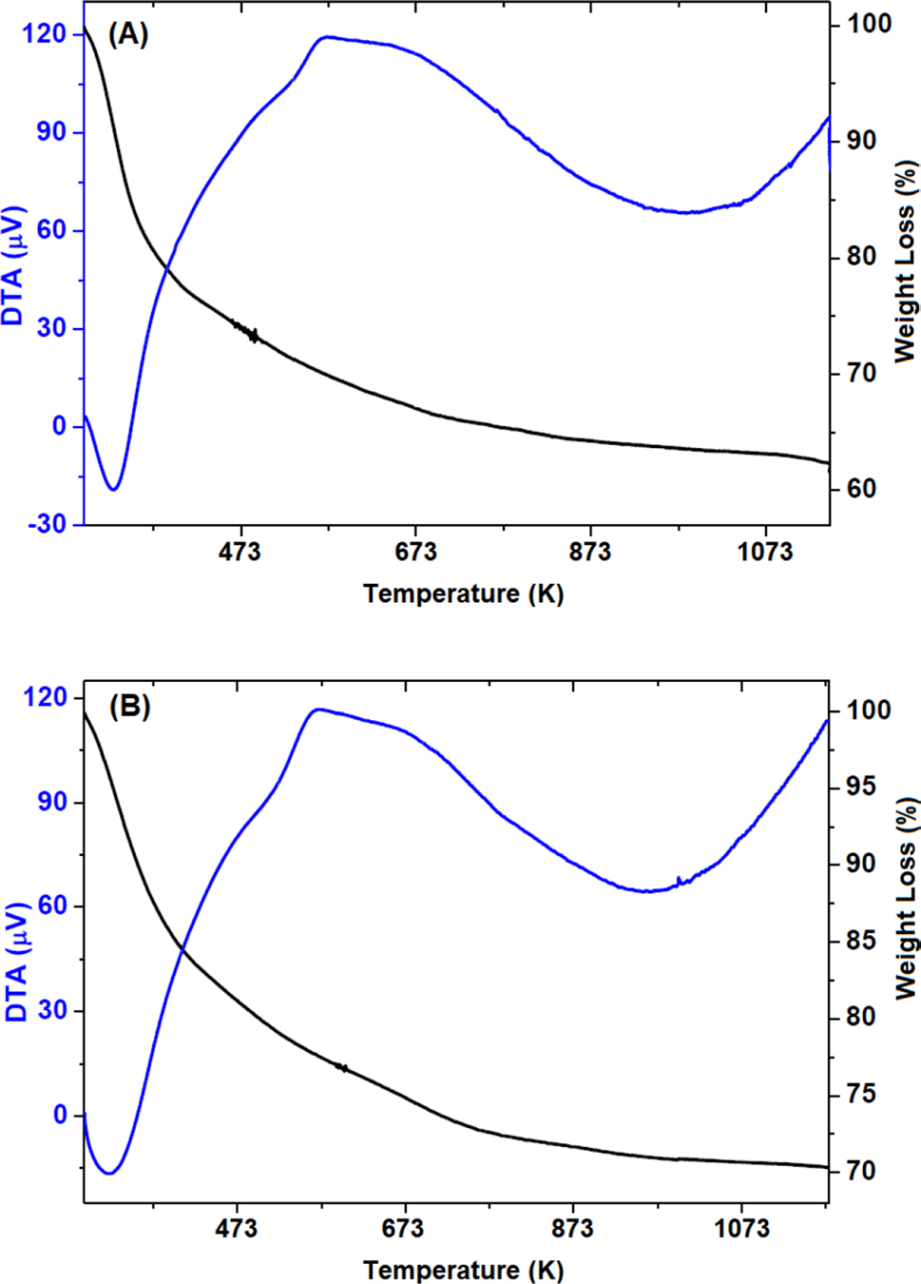
Thermogravimetric analysis of SiTiNb-A **(A)** and SiTiNb-B **(B)**. The graphs show the DTA data (blue line) and the relative weight loss (black line).

The thermal stability of SiTiNb-A and SiTiNb-B materials is evaluated by Thermogravimetric Analysis (TGA-DTA) (Fig. 3). For the SiTiNb-A material (Fig. 3A), in the temperature variation to approximately 383 K, an endothermic event (exo down) is observed with a mass loss of 21%, related to water desorption. From 383 to 528 K (exothermic event, exo up), it was found a mass loss of approximately 8%, due to the dehydroxylation of the groups –SiOH, –TiOH or –NbOH in virtue of structural water loss^37,38^. Above the temperature of 528 K, there was a change in the line referring to DTA, which may indicate an event where there is a change in energy generating a mass loss of approximately 4% (endothermic event, exo down). This event can also be seen in Raman's analysis which will be discussed later. In the range between 673 and 1093 K there is a mass loss also of approximately 4% (endothermic event, exo down) related to the loss of organic matter adhered to the sample^38,39^. Finally, there is a small loss (0.6%) ascribed, possibly, to organic matter aggregated or encapsulated in the pores of the sample^40^, totaling a mass loss of 37.6%.

For SiTiNb-B material (Fig. 3B), the mass losses are similar, totaling 29.7%. Up to 408 K a mass loss of 15.4% is observed (endothermic event, exo down), related to water desorption^37,38^. Between 408 and 523 K the mass loss is approximately 5% (exothermic event, exo up) and in the range of 523 K to 698 K the mass loss also corresponds to 5% (endothermic event, exo down)^38,39^. At 698 K at the end of the process, the mass loss is 4%^38–40^.

**Figure 4:**
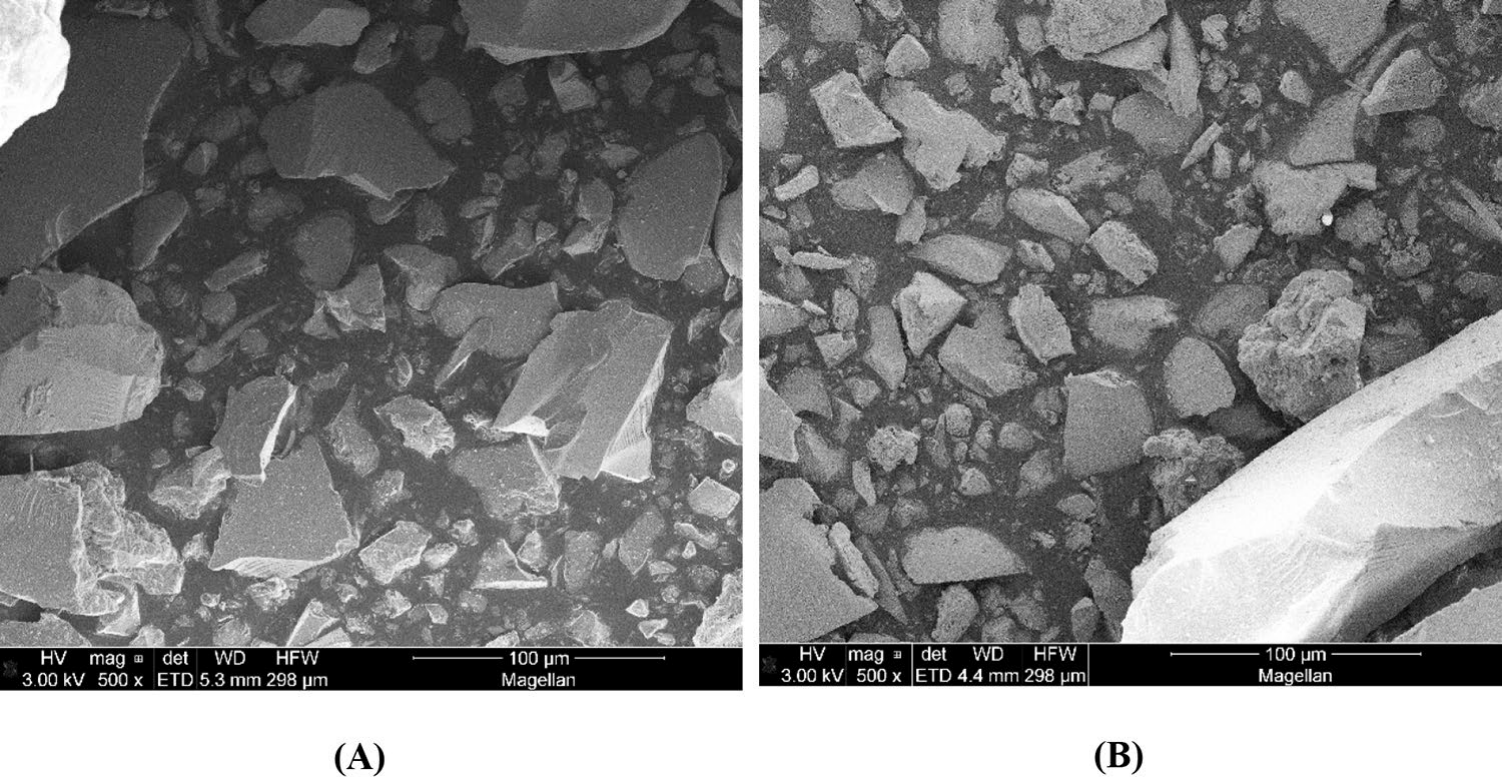
Scanning Electron Microscopy (SEM) micrograph of SiTiNb-A **(A)** and SiTiNb-B **(B)** materials.

As seen in Fig. 4, the Scanning Electron Microscopy (SEM) for SiTiNb-A (Fig. 4A) and SiTiNb-B (Fig. 4B) materials shows the morphology of the particles does not present a uniform size and it presents irregular shape and no-spherical particles. They have been characterized by a flat surface with a rough structure. This characteristic is quite usual for mixed oxides obtained by the sol–gel process^3,4^. Note that the particle size distribution is quite heterogeneous, which is characteristic for mixed oxides obtained by the sol–gel process; and the distribution cannot be estimated by SEM analysis. But there are particles in the vast majority between 10 and 90 µm. Some particles are even larger than 100 µm, but as rods form, they pass through the mesh of the sieve that was used for particles smaller than 90 µm.

**Figure 5:**
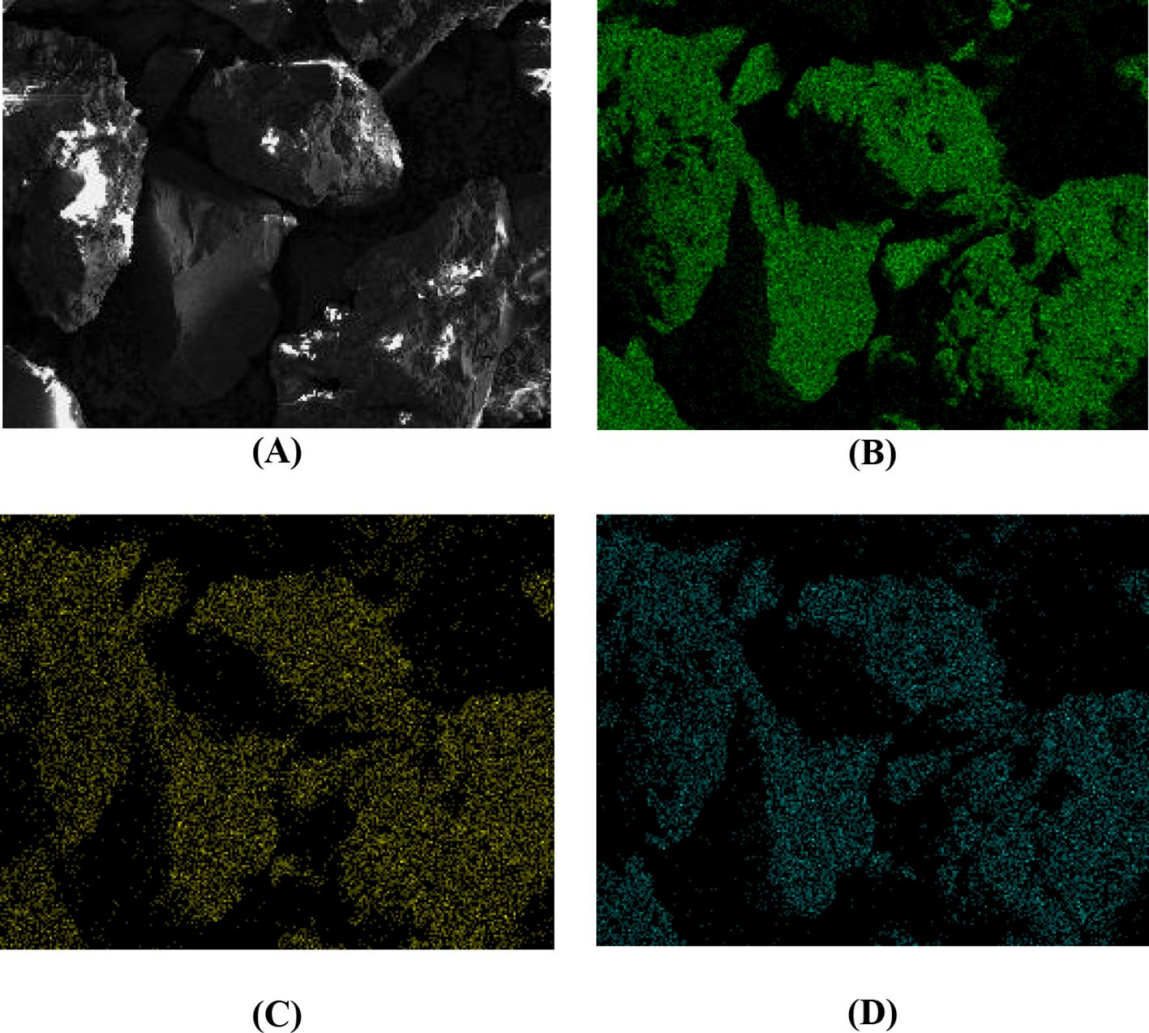
Scanning Electron Microscopy (SEM) micrograph **(A)** and respective Electron Dispersive Spectroscopy images (EDS) of Si **(B)**, Ti **(C)** and Nb **(D)** for SiTiNb-A material. Magnification of 1000x.

The Fig. 5 shows the scanning by EDS analysis of Si (Fig. 5B), Ti (Fig. 5C) and Nb (Fig. 5D) for SiTiNb-A material. The EDS shows the TiO_2_ or Nb_2_O_5_ particles are dispersed without phase segregation or formation of islands at the silica matrix, which was a desired feature. In addition, at the magnification level used (1000x), the SiTiNb-A material seems to be homogeneous with high uniformity on its surface. This dispersion is very important because increases the number of acidic sites on the surface or pores of SiTiNb-A material, and it can be attributed to the strong interactions with siloxane groups of the silica matrix forming strong covalent bonds as the –Si–O–Ti–OH, –Si–O–Nb–OH types, as observed by XRD analyzes.

The percentage values on the surface of the SiTiNb-A material for the elements Si, Ti and Nb were determined from the 3-point analysis by EDS, which were homogeneous, corroborating with the XRF analysis. It was observed through the SEM and EDS analyzes that the SiTiNb-B material presented the same behavior, so the results were not presented.

**Figure 6:**
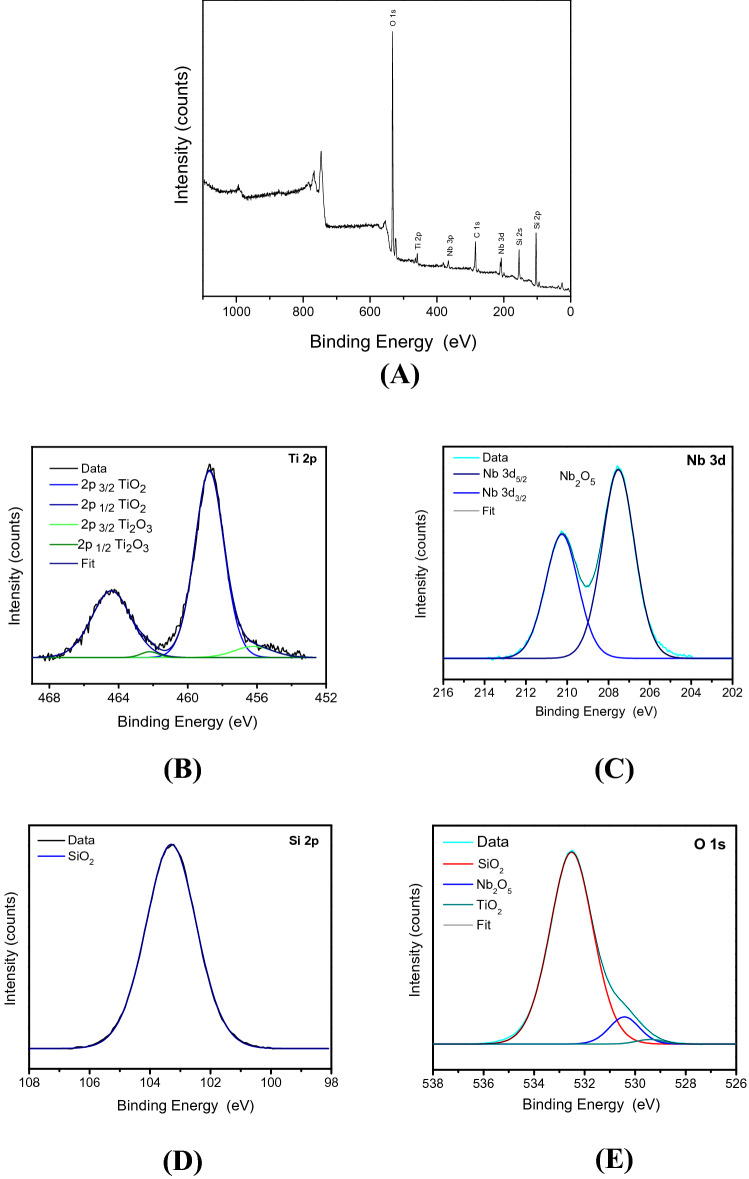
XPS analysis of SiTiNb-A material. Survey Spectrum **(A)** and the higher resolution spectra in the regions of Ti 2p **(B)**, Nb 3d **(C)**, Si 2p **(D)** e O 1 s **(E)**.

**Figure 7:**
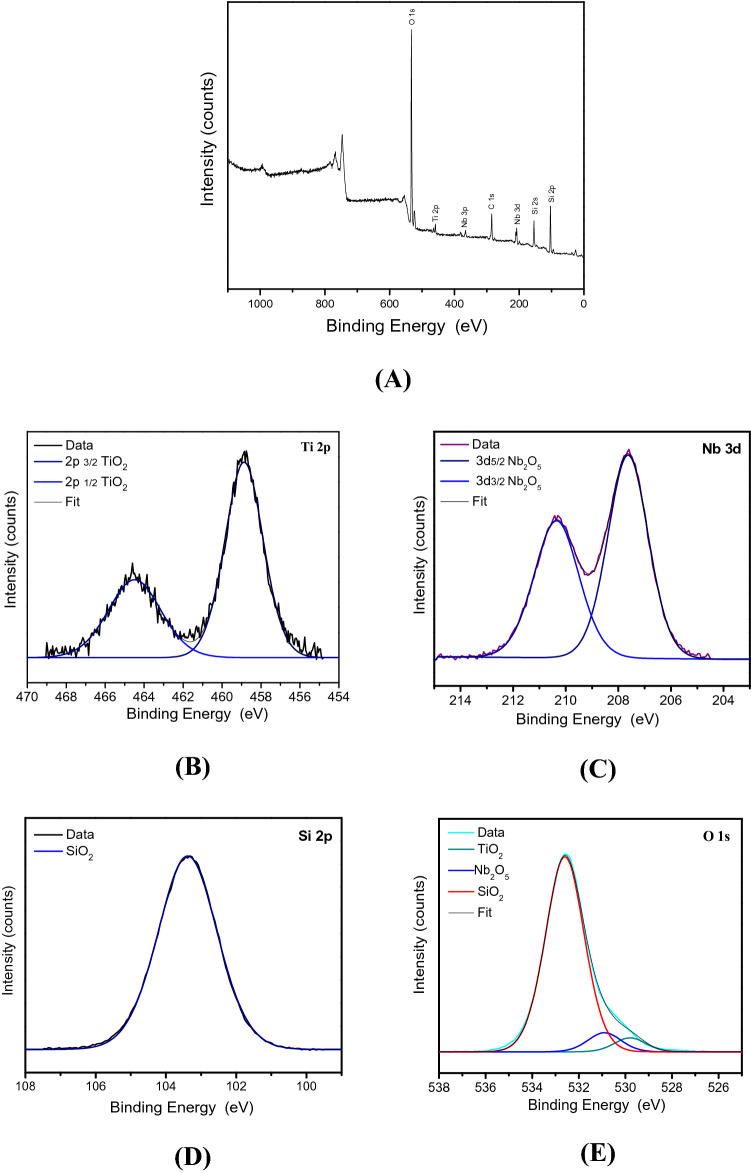
XPS analysis of SiTiNb-B material. Survey Spectrum **(A)** and the higher resolution spectra in the regions of Ti 2p **(B)**, Nb 3d **(C)**, Si 2p **(D)** e O 1 s **(E)**.

The XPS analyses were performed to investigate the chemical states of the elements in the SiTiNb-A and SiTiNb-B materials. The survey spectra (Figs. 6A, 7A) show the presence of O, Ti, Nb and Si elements, which confirm the mixture of SiO_2_, TiO_2_ and Nb_2_O_5_ oxides on the samples surface, also observed in EDS analyses. It was also observed in spectra the presence of residual carbon, accordingly to Barr and Seal^41^ is common on XPS analysis due to the impurities on the air or the material of synthesis, such desorbed CO_2_. The high-resolution spectra in the Ti 2p region for the SiTiNb-A and SiTiNb-B samples (Figs. 6B, 7B), show the peaks at 464.5 and 458.4 eV correspond to the binding energy of components Ti 2p_1/2_ and Ti 2p_3/2_ related to Ti (IV) species in TiO_2_^42^. Furthermore, the sample SiTiNb-A (Fig. 6A) present small peaks in 456.2 and 462.2 eV, according to literature it is related to Ti (III) species in Ti_2_O_3_ analogously to the works of Marino et al.^43–45^.

In the Nb 3d region for SiTiNb-A and SiTiNb-B samples (Figs. 6C, 7C) were observed peaks located at 211.8 and 208.9 eV corresponding to the components 3d_3/2_ and 3d_5/2_ respectively, referring to Nb_2_O_5_ and confirming the presence of Nb (V)^46,47^. The presence of Si (IV) on two samples was confirmed with a centralized peak in 104.7 eV Si 2p region, referring to the Si–O bond in SiO_2_^42,48^ (Figs. 6D, 7D). The O 1 s region showed all oxides in the SiTiNb-A and SiTiNb-B samples composition, SiO_2_, TiO_2_ and Nb_2_O_5_ (Figs. 6E, 7E). The regions in 533.2 eV, 531.0 eV and 530.3 eV were related to the oxygen bonds of the Si(IV), Nb(V) and Ti(IV) species, respectively^46^.

Finally, when comparing the analysis of WDXRF and XPS, where in WDXRF the elements were also quantified in the bulk of the materials, it was observed that there was a higher concentration of Nb_2_O_5_ on the materials surface than in the bulk.

### Spectroscopic characterization

**Figure 8:**
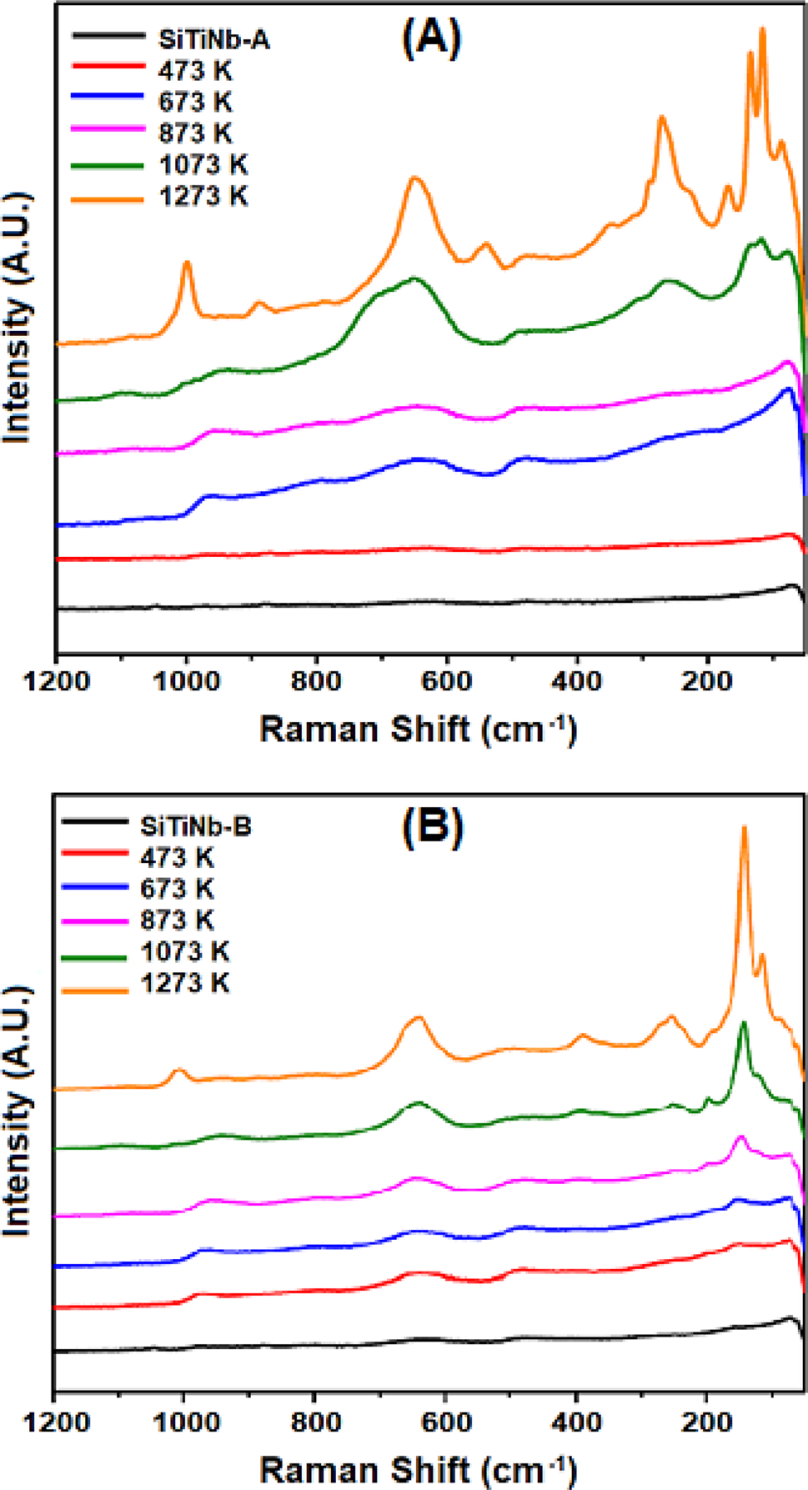
Raman spectra of thermally treated for the SiTiNb-A **(A)** e SiTiNb-B **(B)** materials at different temperatures.

According to the Raman spectra of thermally treated SiTiNb-A (Fig. 8A) and SiTiNb-B (Fig. 8B) materials, there was an appearance of crystalline phase at 1073 K, these bands being present in the sample without calcining and in those calcined at temperatures below 1073 K. With a higher temperature of calcination, this ordering increases and it can also be noticed in the medium and long distance as data observed in the XRD analyzes. According to the Raman spectra of thermally treated samples and the spectroscopic factor group analysis, the anatase phase presented six vibrational modes active in Raman (D_4h_^19^). Thus, for TiO_2_ in anatase form, there were six active Raman modes (3E_g_ + 2B_1g_ + A_1g_), with them being centered at 144 (E_g_), 197 (E_g_), 399 (B_1g_), 513 (A_1g_), 519 (B_1g_) and 639 cm^−1^ (E_g_)^49^. Graça and collaborators^50^ noticed that the Raman displacements for monoclinic Nb_2_O_5_ had better defined bands in samples treated from 1173 K, some of these bands being observed for the materials SiTiNb-A and SiTiNb-B at 1073 K. For both phases, there was an overlap of the bands referring to anatase and niobia in the region of 630–650 cm^−1^. Tables 4 and 5 summarize the Raman displacement values for the most defined peaks of the calcined samples.

**Table 4:** Raman displacement of the highest intensity peaks for the calcined SiTiNb-A samples.

Sample	Higher intensity peaks in Raman (cm^−1^)									
1073 K	–	934	–	651	–	258	–	132	117	77
1273 K	998	–	888	649	538	270	168	133	115	86

**Table 5:** Raman displacement of the highest intensity peaks for the calcined SiTiNb-B samples.

Sample	Higher intensity peaks in Raman (cm^−1^)									
873 K	–	959	–	641	–	–	–	147	–	–
1073 K	–	948	–	639	–	–	196	144	–	–
1273 K	1005	–	–	639	390	253	–	143	115	86

For the samples of SiTiNb-A, it was observed that up to 473 K there was no characteristic peak formation, except for the peak around 80–70 cm^−1^ that appeared in all samples. After 673 K the peaks referring to anatase in 513 (A_1g_), 519 (B_1g_) and 639 cm^−1^ (E_g_)^49^, that appeared as broad bands, shifted to the left and lose definition, while the peaks referring to monoclinic Nb_2_O_5_ appeared with greater definition at 1273 K. The peaks were more evident in 998, 649, 270 and 115 cm^−1^, as described in the literature^50^. Finally, for the samples of SiTiNb-B, there was less definition of the bands in relation to sample A, however, the well-defined peak, at 144 cm^−1^, referring to the anatase, appears at 873 K. Note that in the 150–100 cm^−1^ region there was no overlapping of the bands referring to anatase and niobia.

**Figure 9:**
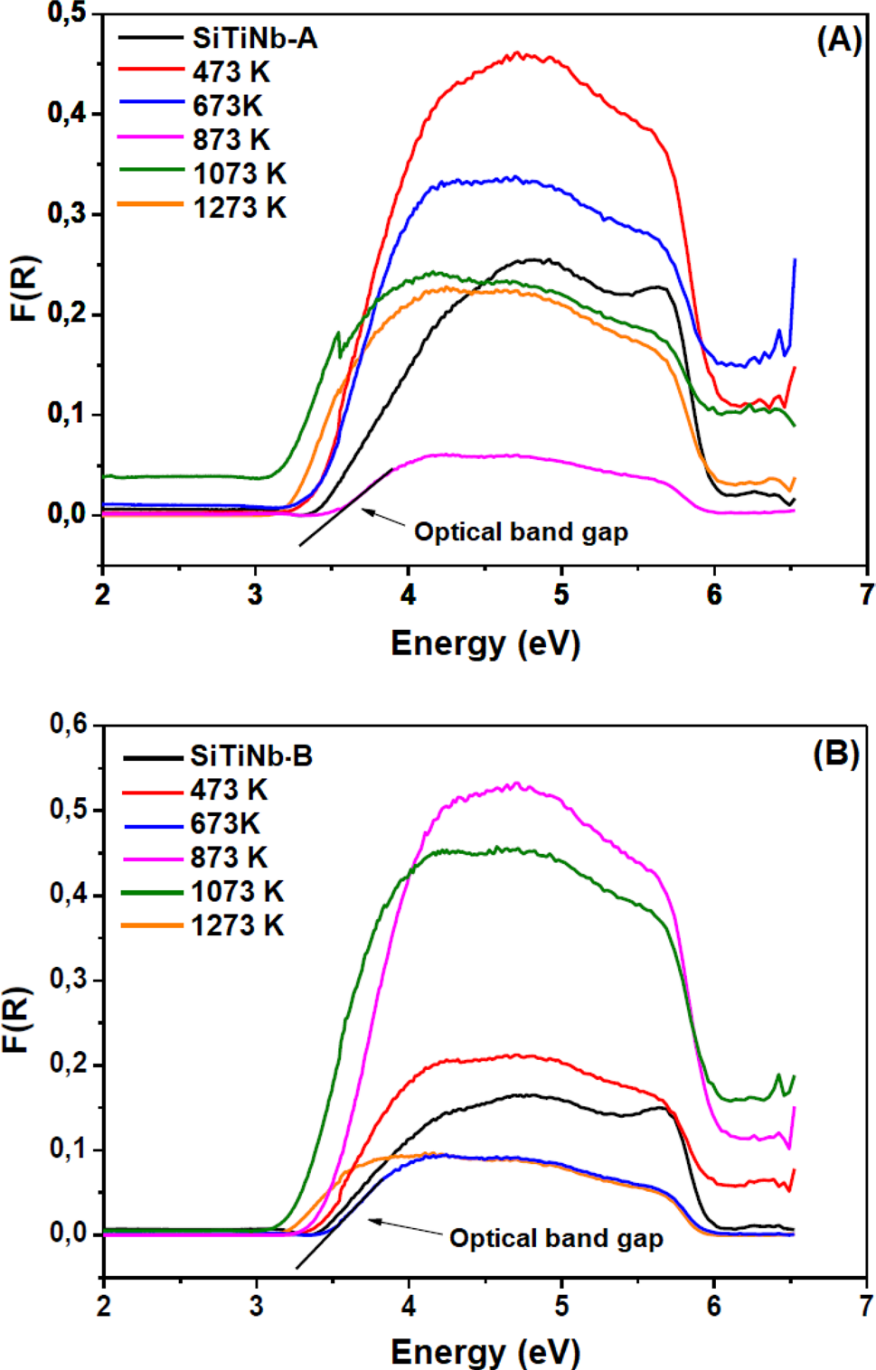
DRS (Kubelka–Munk function, F (R)) and extrapolation of the tangent line to obtain the optical band-gap of the SiTiNb-A **(A)** and SiTiNb-B **(B)** samples treated at different temperatures.

The optical band-gap values were obtained using the Kubelka–Munk (F (R)) function in the Diffuse Reflectance data of the heat-treated samples (Fig. 9) and are shown in Table 6. Nb_2_O_5_ is n-type semiconductor with a band-gap of about 3.4 eV^51^, and that was dependent on oxygen stoichiometry in its structure, varying its band-gap from 3.2 to 4.0 eV. In general, Nb_2_O_5_ had a higher conduction band than TiO_2_ being indicated to obtain open-circuit voltage and efficiency in the conversion of photons. The most thermodynamically stable form of Nb_2_O_5_ is the monoclinic arrangement presented in the synthesized samples^52^. For the TiO_2_ anatase band-gap is observed in the 3.23 eV region^53^; and this transition occurring in anatase can be described as an absorption of the valence band (essentially 2p filled O^2–^) to the conduction band (essentially 3d voids of the Ti).

**Table 6:** Optical band-gap of SiTiNb-A and SiTiNb-B from the function of Kubelka–Munk.

Sample	Optical band-gap (eV)	Sample	Optical band-gap (eV)
SiTiNb-A	3,3	SiTiNb-B	3,3
SiTiNb-A-473	3,3	SiTiNb-B-473	3,3
SiTiNb-A-673	3,3	SiTiNb-B-673	3,4
SiTiNb-A-873	3,5	SiTiNb-B-873	3,4
SiTiNb-A-1073	3,1	SiTiNb-B-1073	3,2
SiTiNb-A-1273	3,1	SiTiNb-B-1273	3,1

The samples which were not calcinated had a band-gap around 3.3 eV and when the appearance of the TiO_2_ anatase and Nb_2_O_5_ monoclinic phases begun, there was a slight increase in the band-gap followed by its reduction after temperatures of 1073 K. Whereas the monoclinic phase stabilizes, it is obtained a band-gap value of 3.1 eV in 1273 K. Thus, the transition from band 2p (full) of oxygen (O^2–^) to band 4d (empty) of Nb^5+^ should occur. It was observed in the spectrum of SiTiNb-A phase a tooth formation (electronic artifact) that masks the proper measurements, requiring deeper analysis to determine minor variations in the band-gap values.

Table 6 shows that the band-gap values did not vary significantly with the different percentages of the crystalline phases. However, in Tables 1 and 2 were shown a variation in the crystallites size of the crystalline phases and a smooth "red-shift" in the energy of band-gap, both in the heat-treated samples.

**Figure 10:**
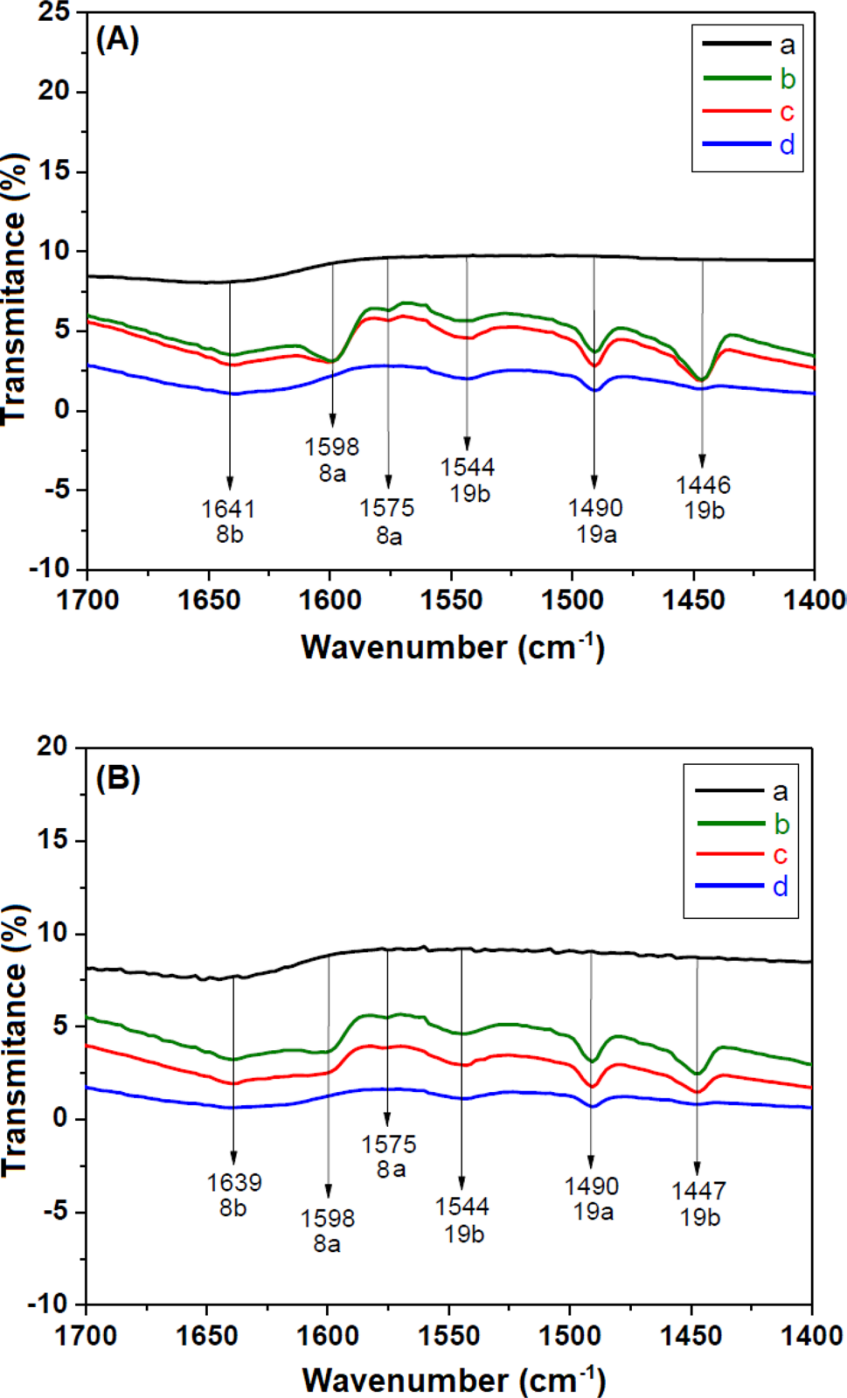
Infrared spectra of pyridine adsorbed on SiTiNb-A **(A)** and SiTiNb-B **(B**) at different temperatures under vacuum: **(a)** pure SiTiNb, **(b)** room temperature, (**c**) 373 K, (**d**) 423 K.

The acid properties of the SiTiNb-A (Fig. 10A) and SiTiNb-B (Fig. 10B) materials were studied using pyridine adsorbed as probe molecule, which is the most used in the literature^54^. The characteristic bands for Lewis and Brønsted acid sites can be observed in both materials: The Lewis acid sites are due to the titanium or niobium ions coordinatively unsaturated^55–58^, and the Brønsted acid sites are a contribution of Ti–OH or Nb-OH groups, for example^11,59–62^.

The weak band at 1575 cm^−1^ is due to the vibrational mode 8a of the pyridine physically adsorbed by weak forces such as van der Waals. Thus, the pyridine molecule exhibits low interaction with the material, and this band disappears whereas the temperature increases^63,64^. The bands at 1598 and 1446 or 1447 cm^−1^, certainly, are due to the 8a and 19b modes of pyridine bonded to the free silanol groups present at the surface of the materials. The –SiOH group of silica matrix by hydrogen bonds seen in the pure SiTiNb ternary oxide treated at room temperature disappears whereas the temperature increases^65^. The band at 1490 cm^−1^ possibly corresponds to the vibrational mode 19a. A band at 1544 cm^−1^ is attributed to the vibrational mode 19b of the pyridine molecule bound to the Brønsted acid sites (–TiOH or –NbOH groups), and this band is not observed for the pure SiO_2_^66^. The 19b mode of pyridine bound to Lewis acid sites is observed as a small shoulder at approximately 1458 cm^−1^ that disappears after heat treatment at 423 K. Finally, the weak band detected at 1641 or 1639 cm^−1^ characterizes the presence of Brønsted acid sites, that decreases while increasing temperature^67^.

In comparison to the SiO_2_/TiO_2_/Sb_2_O_5_ (SiTiSb) material, already described by LaDANM Group^4^, the behavior is different in relation to the SiTiNb material, in this study. It can be reported that in the SiTiSb-A material the presence of the anatase phase is noticed in the sample treated at 473 K. However, in the system with niobium, SiTiNb material, it was only observed the presence of the anatase phase with the appearance of the crystalline oxide phase of monoclinic niobium at 1273 K. This indicates that the SiO_2_/Nb_2_O_5_ network binds more strongly to Ti than the SiO_2_/Sb_2_O_5_ network.

The different structural characteristics and properties showed for the SiTiNb materials with the presence of the Lewis and Brønsted acid sites on its surface made them interesting for use involving application studies, such as adsorbent for metal ions, in photocatalysis, etc. In addition, the band-gap values obtained for both SiTiNb-A and SiTiNb-B show they can be probably used as n-type semiconductors.

## Conclusions

The SiTiNb materials obtained by the sol–gel method, showed a high dispersion of metal oxides at the silica matrix, showed no phase segregation, and presented significant Specific Surface Areas (S_BET_). The XPS analysis indicated the insertion of Ti and Nb atoms in the silica matrix and that antimony is in its higher oxidation state. These materials presented good thermal stability as observed by DRX and TGA‑DTA data. Crystalline phases in the ternary oxides were found only after heating at 1073 K and confirmed by the optical band-gap depend on the oxides concentration and the calcination temperature. The presence of the Lewis and Brønsted acid sites on its surface was confirmed by pyridine test. Due to the characteristics presented of these materials, they are (the most) promising for testing as electrochemical sensors, adsorbent for metal ions and other species in effluents, heterogeneous catalysts and photocatalysis, for instance.

## Supplementary Information

Supplementary Information.
