# Supplementary material for: Study of the characteristics and properties of the SiO2/TiO2/Nb2O5 material obtained by the sol–gel process
Source: Sci Rep. 2021 Jan 13;11:1106. doi: 10.1038/s41598-020-80310-4 (PMC7806853; doi:10.1038/s41598-020-80310-4)
Supplement: Supplementary file 1 — Supplementary Information. [file 41598_2020_80310_MOESM1_ESM.docx]

**Supplementary Material**

**Scientific Reports**

**Study of the characteristics and properties of the SiO_2_/TiO_2_/Nb_2_O_5_ material obtained by the sol-gel process**

Bruna Teixeira da Fonseca^a^, Eliane D’Elia^a^, José Márcio Siqueira Júnior^b^, Sanair Massafra de Oliveira^a^, Kelly Leite dos Santos Castro^a^ and Emerson Schwingel Ribeiro^a*^

*^a^Instituto de Química, Universidade Federal do Rio de Janeiro -UFRJ, CT, Bloco A, Cidade Universitária – Ilha do Fundão, CEP 21941-909, Rio de Janeiro - RJ, Brazil.*

*^b^Instituto de Química, Universidade Federal Fluminense – UFF, Valonguinho, CEP 24020-150, Niterói - RJ, Brazil.*

**^*^**Address correspondence to E-mail: emersonsr@iq.ufrj.br

ORCID: http://orcid.org/0000-0001-8134-0274


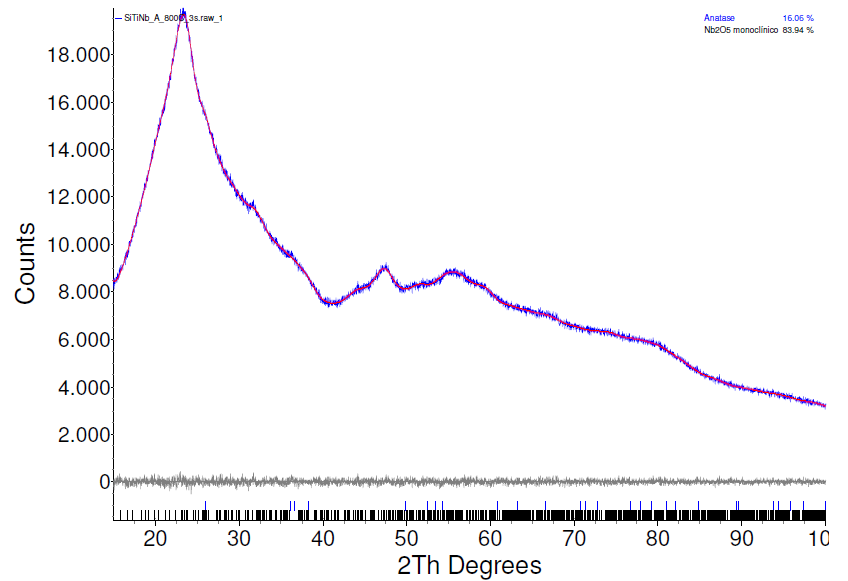


**(A)**

**
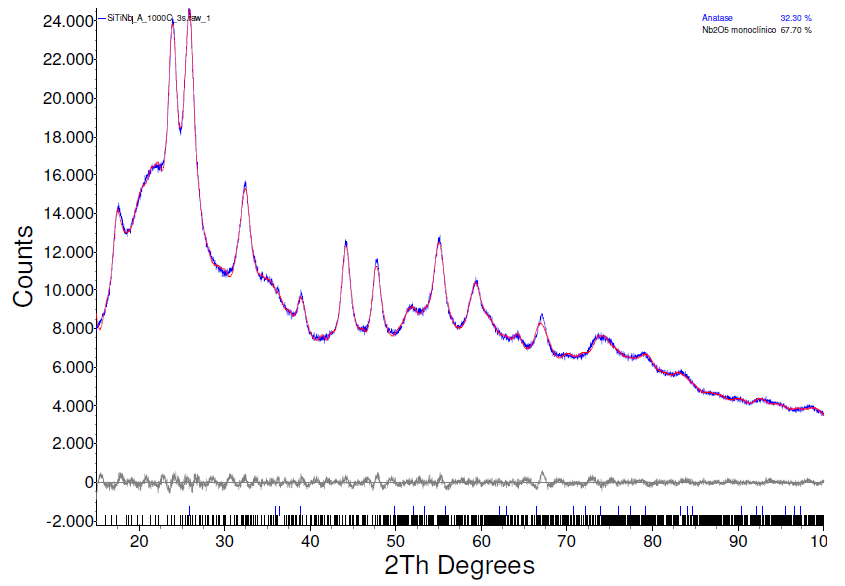
**

**(B)**


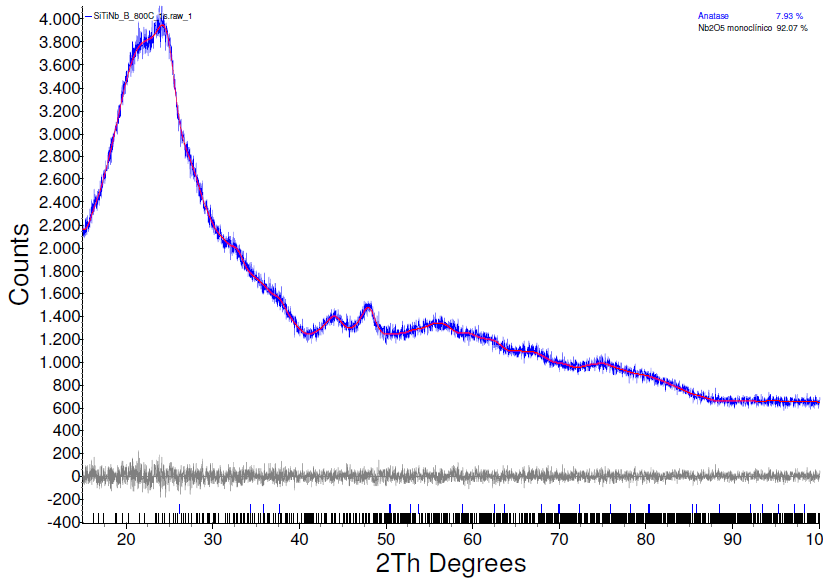


**(C)**

**
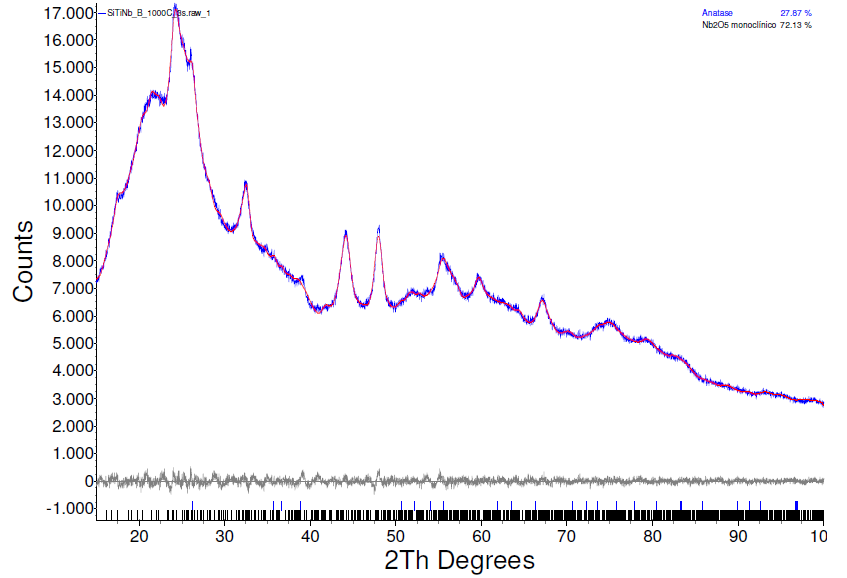
**

**(D)**

**Figure S1.** Refinement by the Rietveld method with the TOPAS Academic V.5 software (Copyright 1992-2012 Alan A. Coelho. Where, for the activation of the program, DLL files are released by Alan) for the materials: SiTiNb-A at 800 ^o^C **(A)**, SiTiNb-A at 1000 ^o^C **(B)**, SiTiNb-B at 800 ^o^C **(C)**, SiTiNb-D at 1000 ^o^C **(D)**. Blue line: experimental data; red line: calculated data and gray line: difference between experimental and calculated data.
